# Insights into the Role of Sialylation in Cancer Metastasis, Immunity, and Therapeutic Opportunity

**DOI:** 10.3390/cancers14235840

**Published:** 2022-11-26

**Authors:** Jianmei Huang, Jianming Huang, Guonan Zhang

**Affiliations:** 1 School of Medicine, University of Electronic Science and Technology of China, Chengdu 610054, China; 2 Biochemistry and Molecular Biology, Sichuan Cancer Institute, Chengdu 610041, China; 3 Department of Gynecologic Oncology, Sichuan Cancer Hospital, School of Medicine, University of Electronic Science and Technology of China, Chengdu 610041, China

**Keywords:** sialylation, sialic acid, cancer, immunotherapy, anti-tumor therapy, metastasis

## Abstract

**Simple Summary:**

Sialylation is the synthetic process of sialoglycans, which are important in tumor transformation, proliferation, metastasis, and immune evasion. The primary subjects of cancer sialylation-related articles over the past decade have been sialylation, cancer, immunotherapy, and metastasis. The interactions of selectins with abnormal sialylated integrins activate endothelial cells and help tumor cells spread. Cancer sialylation conceals tumor antigenic epitopes and suppresses the immunological environment, allowing cancer cells to evade immune surveillance. Targeting tumor-derived sialoglycans may be an effective cancer therapeutic strategy for limiting tumor cell spread, revealing immunogenic tumor antigens, and boosting anti-cancer immunity.

**Abstract:**

Sialylation is an enzymatic process that covalently attaches sialic acids to glycoproteins and glycolipids and terminates them by creating sialic acid-containing glycans (sialoglycans). Sialoglycans, usually located in the outmost layers of cells, play crucial biological roles, notably in tumor transformation, growth, metastasis, and immune evasion. Thus, a deeper comprehension of sialylation in cancer will help to facilitate the development of innovative cancer therapies. Cancer sialylation-related articles have consistently increased over the last four years. The primary subjects of these studies are sialylation, cancer, immunotherapy, and metastasis. Tumor cells activate endothelial cells and metastasize to distant organs in part by the interactions of abnormally sialylated integrins with selectins. Furthermore, cancer sialylation masks tumor antigenic epitopes and induces an immunosuppressive environment, allowing cancer cells to escape immune monitoring. Cytotoxic T lymphocytes develop different recognition epitopes for glycosylated and nonglycosylated peptides. Therefore, targeting tumor-derived sialoglycans is a promising approach to cancer treatments for limiting the dissemination of tumor cells, revealing immunogenic tumor antigens, and boosting anti-cancer immunity. Exploring the exact tumor sialoglycans may facilitate the identification of new glycan targets, paving the way for the development of customized cancer treatments.

## 1. Introduction

Sialylation is a type of glycosylation that involves the covalent addition of sialic acid to the terminal glycans of glycoproteins and glycolipids. This enzymatic process is tightly regulated by sialyltransferases (STs) and sialidases/neuraminidases (NEUs) [1,2]. Based on the category of glycosidic linkage, sialylation can be classified into three types: α2-3-, α2-6-, and α2-8-sialylation. Sialic acids usually terminate glycans to form the outmost layers of cells and thus play crucial roles in cellular functions [2,3,4]. They were first proposed as biomarkers and potential therapeutic targets for cancers, following the discovery of the elevation of total sialic acids (both glycoprotein/glycolipid-bound and free sialic acids) in cancer cells in the 1960s [5]. Aberrant sialylation is one of the universal features of cancer and plays biologically important roles in tumor transformation, growth, metastasis, and immune evasion [4,6].

Aberrant sialylation is both a consequence of tumor transformation and a driver of the malignant phenotype [2,7]. Tumorigenesis is driven mainly by genomic variations that induce oncogene activation and/or tumor suppressor inactivation. These genomic variations may also induce altered sialylation and its related enzyme expression. Oncogenes, such as Ras and c-Myc, are reported to regulate the expression of STs and increase the sialylation of corresponding proteins/antigens, resulting in tumor aggressiveness [7]. The upregulation of STs of tumor cells is usually associated with tumor proliferation, distant metastasis, advanced stages, and shorter recurrence-free intervals [8,9,10,11].Epidemiologically, cancer is one of the major causes of premature death globally and is expected to become the leading killer [12]. In 2020, there were an estimated 19.3 million new cases and 10 million cancer deaths worldwide. If the national rates estimated in 2020 persist, the number of new cases will grow by 47% in 2040 [13]. With the burden growing, cancer has become a major concern for public health. Consequently, it is necessary to develop novel interventions to reduce cancer mortality. Sialic acids impact almost all aspects of tumors, especially their imperative roles in immune modulation, suggesting related therapeutic opportunities for cancers. Therefore, we reviewed articles on sialylation in cancer, covering their latest breakthroughs and significant issues that are involved in cancer metastasis, anti-cancer immunity, and anti-cancer therapy. This review not only supports the concept of interfering with sialylation as a novel, potentially anti-cancer strategy, but also demonstrates that identifying altered sialylation may enrich the tumor glycome, reveal potential glycan targets, and pave the way for patient-tailored cancer treatments.

## 2. Overview of Articles on Sialylation in Cancer

**Figure 1:**
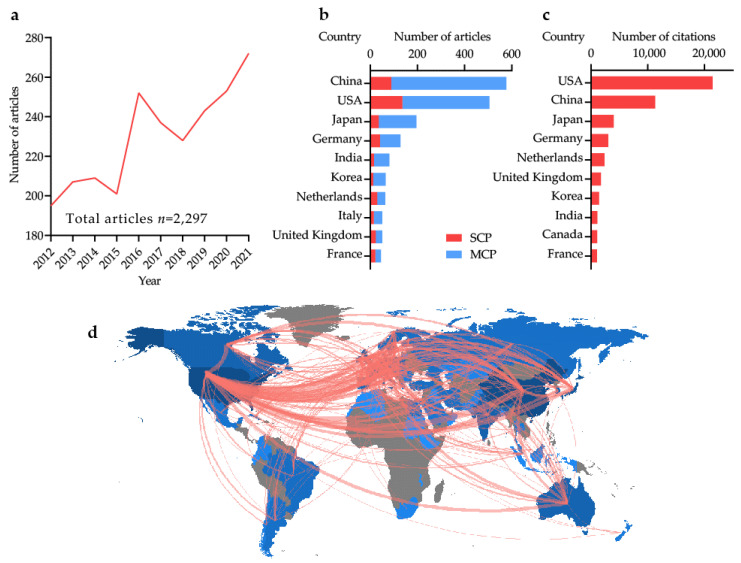
Global trends in publications on cancer sialylation: (**a**) the annual publications over the past decade; (**b**) top 10 countries with the most articles. SCP: single country publications; MCP: multiple country publications; (**c**) top 10 countries with the most total citations of related articles; and (**d**) world map showing collaborations between different countries in this field. The figures were plotted automatically using the bibliometrix package in R version 4.2.0 based on the retrieved articles.

Using bibliometric analysis [14], we summarized cancer sialylation-related literature from the Web of Science (WoS) Core Collection over the last decade. The search strategy was (TS=(sialic acid OR sialylation OR sialoglycan)) AND (TS=(cancer OR tumor OR malignancy OR carcinoma OR neoplasm)). A total of 2,297 articles on sialylation in cancers were retrieved on 12 September 2022 (excluding reviews and irrelevant articles; Figure 1a). The number of articles showed a fluctuating upward yearly trend, and especially consistent growth throughout the past four years (Figure 1a). Total annual articles increased by 48.63% (from 195 to 272), with a yearly growth rate of 4.50%. (Figure 1a). China contributed the most articles and single-country publications (SCP; Figure 1b). The United States of America (USA) exhibited the highest multiple-country publications (MCP; Figure 1b). Studies from the USA had the highest number of citations, followed by those from China (Figure 1c). World collaborations between the USA and China were the most frequent, followed by those between the USA and Germany (Figure 1d).

**Figure 2:**
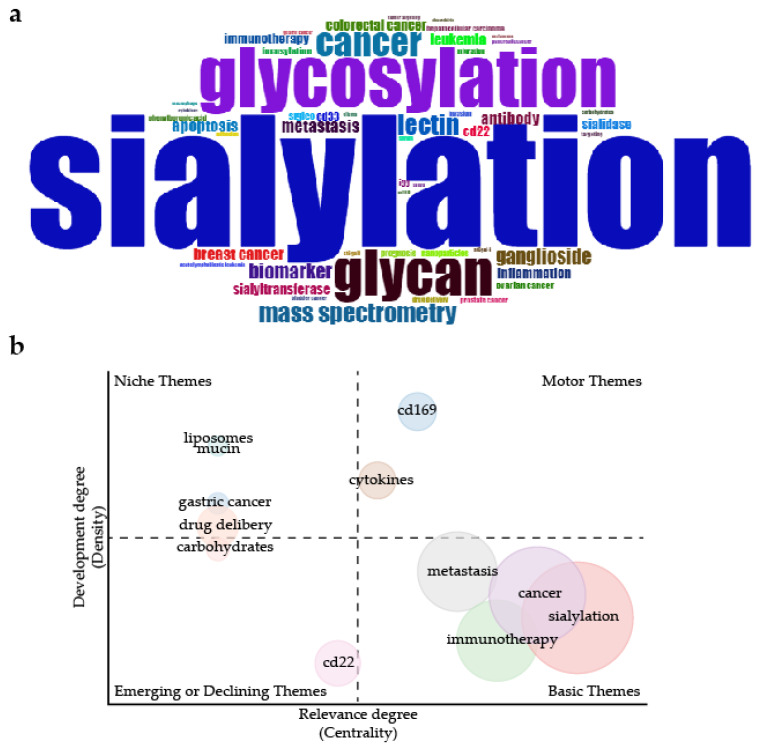
Keywords and topics analysis of articles about cancer sialylation: (**a**) WordCloud showed the top 50 most frequent words. The frequency of keywords determined the font type; and (**b**) thematic map plotted by the authors’ keywords. Both (**a**,**b**) were plotted automatically using the bibliometrix package in R version 4.2.0 based on the authors’ keywords in the retrieved articles.

The global increase of cancer sialylation-related articles indicates their crucial biological significance. Reviewing the top 10 most-cited articles (Table 1), we found that sialylation has a broad impact on various aspects of tumor biology, including immunosurveillance [15,16], stemness [10], migration and progress [11,17,18], chemosensitivity [19,20], and targeted therapy [11,21]. The top five most frequent words in the authors’ keywords of all the retrieved articles were “sialylation”, “glycosylation”, “glycan”, “cancer”, and “lectin” (Figure 2a). The thematic map in Figure 2b shows that sialylation (510 occurrences), cancer (277 occurrences), immunotherapy (145 occurrences), and metastasis (136 occurrences) were the most popular research topics. We will then go over these themes.

## 3. Structural Basis of Sialylation

**Figure 3:**
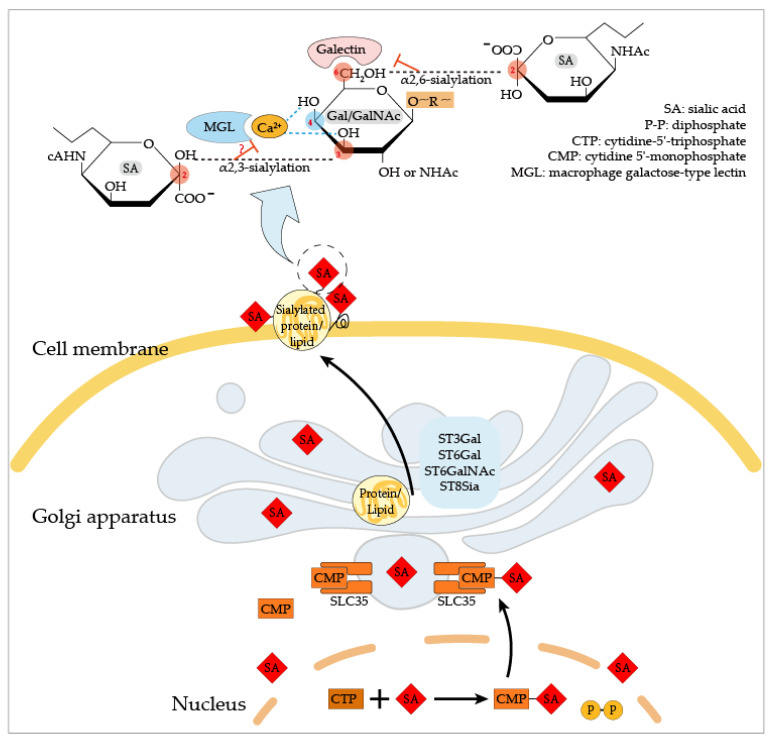
The structural basis and process of sialylation.

Sialylation is the enzymatic process by which sialic acid is attached to galactose (Gal), N-acetyl galactosamine (GalNAc), or another sialic acid. The activated form of sialic acid, cytidine 5′-monophosphate-sialic acid (CMP-Neu5Ac), is synthesized by CMP-Neu5Ac synthase in the cell nucleus [23]. The cytidine-5′-triphosphate (CTP) and Neu5A bind to the CMP-Neu5Ac synthase mononucleotide-binding pocket and Neu5Ac-binding pocket, respectively, forming a sialyl monophosphate diester bond between the C-2 of Neu5Ac and the α-phosphate of CMP and a free diphosphate and reverting the CMP-Neu5Ac synthase to an open state [24]. As a donor, CMP-Neu5Ac is transported to the Golgi apparatus. And this transport can be nearly entirely inhibited by 5′CMP [25]. It is later confirmed that the transport of sialic acid is modulated by the recognition of CMP, whereas “free” sialic acid cannot reach the binding pocket [26]. The CMP-Neu5Ac transporter (CST), a solute carrier SLC35 family member, is the rate-limiting factor of sialylation. CST transfers CMP-Neu5Ac into the Golgi lumen while also antiporting CMP into the cytoplasm [24,27] (Figure 3). The nucleobase pocket of CST utilizes Y214 of SLC35A1 and G239 of SLC35A2 to distinguish cytosine from uracil [28]. Since CMP is an inhibitor of ST activity, the compartmentalization of CST and STs in the Golgi apparatus ensures efficient sialylation [27].

Based on substrates and glycosidic linkages, STs can be divided into four categories: ST3Gal1-6, ST6Gal1-2, ST6GalNAc1-6, and ST8Sia1-6. The first three groups of enzymes catalyze the formation of α2-3 and α2-6 linkages between sialic acids and Gal or GalNAc, that is, sialylation of Gal/GalNAc usually occurs at the 3- or 6-hydroxyl. However, 6-hydroxyl is required for galectin binding [29]. The 3- and 4-hydroxyl groups of Gal/GalNAc are essential for calcium chelating when bound to macrophage galactose-type lectin (MGL) [30,31]. Since it has been demonstrated that MGL can interact with Sialyl Tn (STn, NeuAcα2,6GalNAc-Ser/Thr), it appears that α2-6-sialylation of GalNAc has no effect on MGL binding [32,33].Thus, α2-3-sialylation of GalNAc may inhibit MGL binding (Figure 3). Galectin-dependent cell behaviors, such as galectin-3-induced apoptosis, may be affected by α2-6-sialylation [29,34], MGL-dependent cell behaviors, such as MGL-mediated clearance, may be affected by α2-3-sialylation [35]. The 4-, 8-, and 9-hydroxyl, N-acetyl methyl, amide, and carboxyl groups of sialic acid contribute to the binding of complement factor H and α2,3-sialic acid terminated glycans (Neu5Acα2-3Galβ1-4Glc), resulting in complement evasion [36]. Furthermore, the polysialic acids (polySia) synthesized by ST8Sias form steric and electrostatic exclusions of the acell surface, resulting in anti-adhesive properties and mediating recognition patterns [37,38].

## 4. Sialylation and Cancer Metastasis

Metastasis is a multi-step, inefficient process that accounts for approximately 90% of cancer-related deaths [39,40,41]. The process of cancer metastasis can be briefly summarized as cancer cells escaping their initial sites, surviving in blood and lymph transfer, and developing new distal tumor sites. Sialylation modifies the conformation of essential proteins to promote cancer cell proliferation, invasion, and migration [42]. α2-6-Sialylation of epidermal growth factor receptor (EGFR) regulates the epithelial-to-mesenchymal transition (EMT) of cancer cells [43] and sustains its membrane retention, regulating integrin tension, focal adhesion, and cell motility [44,45]. Moreover, tumor migration and invasion are promoted by increased adhesion to collagen I via α2-6-sialylation of β1 integrin [46], although hyposialylated β1 integrin is reported to enhance the binding of myeloid cells to fibronectin [47] and colonocytes to collagen I [48]. α2,3-Sialylated CD44 improves adhesion to hyaluronic acid, hence boosting cancer cell motility and metastasis [49]. α2-6-Sialylation of fibroblast growth factor receptor (FGFR) increases the cellular signaling mediated by extracellular regulated protein kinases 1/2 (ERK1/2) and focal adhesion kinase (FAK), thus promoting cancer cell migration [50]. Besides, sialylated tumor-associated carbohydrate antigens, such as STn, are reported to increase cancer invasion and are associated with a poor prognosis [51,52]. In order to survive the bloodstream and lymphatic transport, cancer cells upregulate ST6GAL1 to increase α2-6-sialylation of Fas and tumor necrosis factor receptor 1 (TNFR1), which inhibits apoptotic signaling and ensures the formation of the secondary tumor site [53].

**Figure 4:**
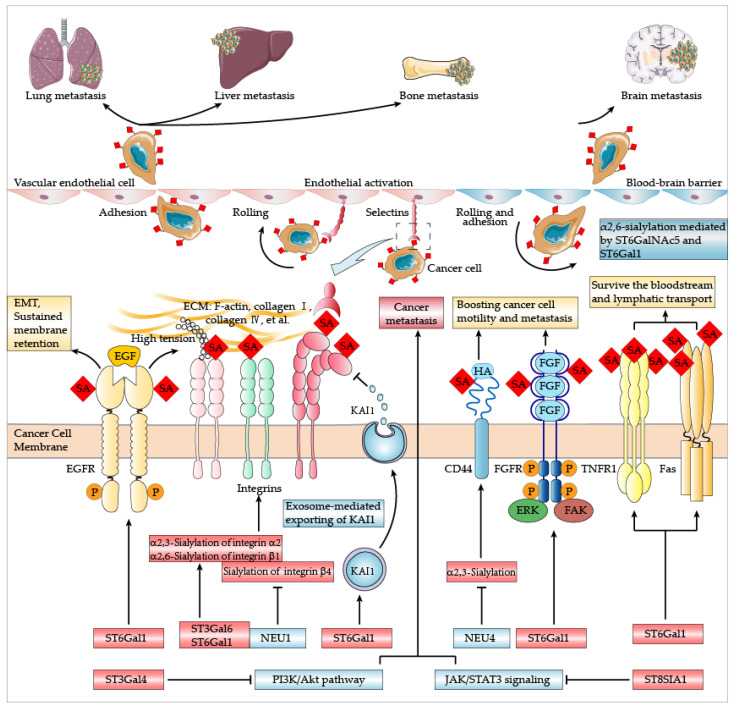
The impact of sialylation on tumor metastasis. α2,6-Sialylation of EGFR sustains its membrane retention and regulates the EMT of cancer cells, enhancing integrin tension, focal adhesion, and cell motility [43,44,45]. α2,3-Sialylation and α2,6-sialylation of integrins enhance their adherence to ECM [46,57,58]. α2,3-Sialylation of CD44 improves adhesion to HA, hence boosting cancer cell motility and metastasis [49]. α2,6-Sialylation of FGFR increases the ERK1/2-FAK signaling to promote the migration of cancer cells [50]. Furthermore, α2-6-sialylation of Fas and TNFR1 inhibits apoptotic signaling and helps the survival of bloodstream and lymphatic transport, consequently ensuring the formation of the secondary tumor site [53]. However, some labs have found that STs or sialylations play some opposite roles in cancer invasion and spread. For example, ST6Gal1 promotes the exosome-mediated export of the metastasis suppressor KAI1, which inhibits integrin signaling [59]. ST3Gal4 and ST8SIA1 are also reported to suppress the metastasis of cancer cells by inhibiting PI3K/Akt pathway and the phosphorylation of JAK2/STAT3, respectively [60,61]. SA: sialic acid, HA: hyaluronic acid, EGFR: epidermal growth factor receptor, EGF: epidermal growth factor, EMT: epithelial-to-mesenchymal transition, FGFR: fibroblast growth factor receptor, FGF: fibroblast growth factor, P: phosphorylation, TNFR1: tumor necrosis factor receptor 1, ERK: extracellular regulated protein kinases, FAK: focal adhesion kinase, ECM: extracellular matrix, KAI1: Kang-Ai 1, NEU: neuraminidase, PI3K/Akt: phosphoinositide-3 kinase/serine/threonine protein kinase B, JAK2/STAT3: Janus kinase 2/signal transducer and activator of transcription 3.

Moreover, tumor cell migration in a hypoxic environment is maintained by polySia [54]. The hypoxic microenvironment increases the polysialylation of neural cell adhesion molecule (NCAM), which results in increased glioblastoma cell motility [55]. Furthermore, steric hindrance caused by cell surface polySia confers the anti-adhesive property to polySia-positive tumors and facilitates metastatic or invasive growth [37]. Overall, altered sialylation is involved in the spread of some malignancies [56] (Figure 4).

### 4.1. Lung Metastasis

During the formation of pulmonary metastasis, interactions between P/L-selectin and blood components permit and initiate the formation of a metastatic milieu, while E-selectin is responsible for the local stimulation of lung microenvironment endothelial cells. Reduction of P/L-selectin, not E-selectin, significantly reduces lung metastasis [62]. However, E-selectin upregulates FAK in lung vasculature, hence facilitating the homing of cancer cells to lung [63]. Selectins also engage in endothelial activation caused by complicated interactions between tumor cells, platelets, and leukocytes, and then upregulate the expression of C-C chemokine ligand 5 (CCL5), which promotes the survival of tumor cells and leads to local lung metastasis [64]. Through these cancer cell stimulations, CCL5 derived from mesenchymal stem cells further boosts cancer cells’ motility, invasion, and metastasis [65]. Additionally, E-selectin interacts with its high-affinity ligands, sialyl Lewis X (sLeX) and sialyl Lewis A (sLeA), increasing cancer cell adherence and lung metastasis [66]. As ligands of selectins, sialic acids contribute to the negative charges of tumor cell surfaces. Cancer cell motility can be reduced by neutralizing the negative charges caused by sialoglycans overexpressed on cancer cells while keeping the glycan moieties [67]. The sialic acid blockade has been reported to reduce lung metastasis in a murine melanoma model [68]. These studies indicate that interrupting the process of sialylation shows the potential to reduce lung metastasis and improve the outcomes of cancer patients.

### 4.2. Liver Metastasis

Tumor-associated mucins typically have shortened core structures and sialylated epitopes [69]. Sialyl Tn (STn) on MUC5AC synthesized by ST6GalNAc1, which is upregulated by mutant p53^R175H^, promotes the liver metastasis of lung cancer [70]. Sialylated structures of MUC16 bind to E- and L-selectin to promote the metastatic ability of pancreatic cancer cells [71]. The interaction of MUC1 and galectin-3 promotes cancer spread by altering tumor cell surface polarization and increasing the exposure of cell surface adhesion molecules [72]. MUC1-positive gastric cancer cells with sialyl Tn (sTn) antigen exhibit more metastatic potential than MUC1-positive cells alone [52]. MUC13 is upregulated in the liver metastasis of metastatic colon cancers, indicating its role in cancer migration and metastasis [73].

Additionally, sialylation of integrin β4 enhances FAK and ERK1/2 pathway signaling to facilitate the liver metastasis of colon cancer cells [57]. The activation of ERK by E-selectin enhances the extravasation and transendothelial migration of colon cancer cells by activating Src kinase and dissociating the VE-cadherin/beta-catenin complex [74]. E-selectin enhances the adhesion of cancer cells to the sinusoidal endothelial cells, promoting metastasis to the liver [75]. Thus, liver metastasis of cancer cells is mucin- or E-selectin-dependent. Understanding the mechanisms of liver metastasis may pave the way for overcoming the relevant cancer therapeutic obstacles.

### 4.3. Bone Metastasis

Bone vascular niche E-selectin binding with disseminated tumor cells triggers both their mesenchymal-epithelial transition (MET) and cancer stem cell traits [76]. E-selectin interacts with sLeX and sLeA involved in EMT of colon cancer cells [77]. Breast cancer cells enter bone sinusoidal niches via E-selectin contacts and are anchored by stromal cell-derived factor 1/C-X-C chemokine receptor type 4 (SDF1/CXCR4) interactions to develop dormant micrometastases [78]. The sLeX of estrogen receptor alpha-positive breast cancer contributes to its bone metastasis [79]. Breast cancer stem cells also exploit sialic acid interactions to induce immune tolerance and distant metastasis [80]. In bone metastatic prostate cancer cells, α2,3-sialylation of α2 subunit of integrin α2β1 is upregulated and plays a crucial role in their initial adhesion capacity [58]. Furthermore, the bone-homing behavior, adhesion, and migration of multiple myeloma cells are also impacted on α2,3-sialylation regulated by ST3Gal6 [81]. According to the roles of sialylation in MET, EMT, and adhesion capacity, sialylation is crucial for the initiation of bone metastasis. Additionally, medicinal approaches that interfere with sialylation may hold tremendous promise for preventing bone migration.

### 4.4. Brain Metastasis

High sialylated *N*-glycans facilitate breast cancer cells transit through the blood-brain barrier, hence promoting breast cancer brain metastasis [82,83,84]. ST6GalNAc5 and ST6Gal1, which mediate α2,6-sialylation, are upregulated in the brain metastasis process of breast cancer [84,85]. The expression of ST6GalNAc5 in breast cancer cells enhances their ability to adhere to brain endothelial cells and breach the blood-brain barrier [85]. Similarly, α2,6-sialylated 4G8 (an IgG antibody drug) affects blood-brain barrier penetration via competitively inhibiting neonatal Fc-receptor-mediated transport [86]. Therefore, sialylation of the cell surface plays a role in breaching the blood–brain barrier and subsequent colonization, and its disruption offers therapeutic opportunities.

### 4.5. Controversial Roles in Metastasis

Interestingly, sialylation has been shown in some labs to play distinct roles in cancer invasion and spread. Although sialyl-Tn is associated with a poor outcome for breast cancer [51,87,88,89], ST6GalNAc2 was identified as a metastasis suppressor of breast cancer by in vivo RNA interference (RNAi) screen combined with next-generation sequencing [90]. High ST6GanNAc2 in estrogen receptor (ER)-positive breast cancers reduces galectin-3 binding and metastasis by increasing the sialylation of core 1 antigen, whereas low ST6GanNAc2 in ER-negative breast cancers shows high endothelial cell adhesion and metastasis via galectin-3 binding [91]. These suggest caution when using ST6GalNAc2 as a possible biomarker for predicting metastases in ER-negative breast cancers [90]. Moreover, sialic acid-containing GM3 has been reported to reduce phosphoinositide-3 kinase/serine/threonine protein kinase B (PI3K/Akt) signaling to increase breast and colon cancer migration and invasion via inhibiting EGFR phosphorylation, upregulating phosphatase and tensin homolog (PTEN) expression, and interacting with integrins [92]. On the contrary, downregulation of ST3Gal4 is associated with malignant progression [60,93] in part by activating PI3K/Akt pathway in renal cell carcinoma [60]. Similarly, certain ganglioside modifications (including the increase of GM3) mediated by ST6Gal1 and ST6GalNAc5 can inhibit glioma invasion [94]. In addition, ST6Gal1 promotes the exosome-mediated exporting of the metastasis suppressor Kang-Ai 1 (KAI1, also known as CD82), thereby reducing KAI1-mediated suppression of integrin signaling in human metastatic colorectal cancer cells [59]. In bladder cancer, ST8SIA1 is reported to decrease proliferation, invasion, and migration by inhibiting the phosphorylation of JAK2 and STAT3, thus downregulating their target genes’ transcription [61].

These conflicting roles of STs in cancer invasion and metastasis may excite the interests of researchers and may pave the way for the uncovering of underlying mechanisms. Prevailing opinions hold that sialylation of cell membrane proteins/lipids increases tumor spread. However, it has been found that highly expressed STs hinder cancer invasion and migration via inhibiting the PI3K/Akt and JAK2/STAT3 pathways [60,61]. The downstream signaling of PI3K/Akt is activated by the phosphorylation of two key conserved sites (Thr308 and Ser473) of Akt [95,96]. Since O-GlcNAcylation, adds β-D-N-acetylglucosamine to protein serine or threonine residues, O-GlcNAcylations at or around Thr308 and Ser473 may compete with phosphorylation and exert distinct effects. The O-GlcNAcylations at Thr305 and Thr312 are reported to inhibit the phosphorylation at Thr308, by disrupting its interaction with phosphoinositide-dependent kinase 1 (PDK1) [97]. The increased O-GlcNAcylation at Ser473 hinders its phosphorylation and enhances the apoptosis of murine pancreatic β cells [98]. O-GlcNAc can be identified through galactosylation and then sialylation [99,100]. O-GlcNAcylation regulates the sialylation of glycosphingolipids and impacts cell-cell interactions as well as signal transduction in human non-pathogenic or cancerous colon cells [101]. How specific sialylation levels and glycoforms trigger signal transduction to switch on/off cancer spread remains a mystery. Overall, sialylation, as a type of post-transcriptional modification, is sure to affect the protein functions; yet its complex impacts on cancer invasion and metastasis are still obscure.

## 5. Sialylation and Cancer Immunity

### 5.1. Immune Recognition

Sialic acids usually occupied the terminal position of glycan chains, making them important mediators for interaction with the surrounding environments. Thus, they play vital roles in anti-recognition biological masks and are essential for self-recognition by self-associated molecular patterns (SAMPs) [102,103,104]. The dense sialoglycan coating on the tumor cell surface forms steric and electrostatic exclusion, masking underlying glycans and protein epitopes to avoid cell recognition [38]. Sialoglycans upregulated in malignancies have been considered “antigenic masking” on tumor cell surfaces [105,106,107,108]. Glycocalyx coats profoundly affect tumor immunogenicity or conceal tumor-associated antigens that are considered deficient [109]. Moreover, sialic acids, located in the outmost layer of tumor cells, mask neoantigens which can be recognized by the immune system [106,107]. Similar to the immune evasion mechanisms of some microbes, tumor cells also decorate themselves with sialic acid-binding antigens as a “don’t eat me” signal to evade host immunity [110,111].

Tumor antigens are typically weak immunogenic. Heavy glycosylation of tumor-derived MUC1 causes its long-term retention in early endosomes without degradation, posing a barrier to DC presentation [112]. It has been demonstrated that glycosylation of tumor-associated proteins influences antigen processing and/or epitope composition. Antigen epitopes are unique for glycosylated and deglycosylated proteins [113,114]. Moreover, the sialylation of therapeutic antibodies is believed to reduce immunogenicity by eliciting tolerogenic effects at the level of DC recognition and uptake [115]. Therefore, emerging approaches for increasing tumor cells’ immunogenicity may lie in altering their glycan structures.

### 5.2. Anti-Cancer Immunity Modulation

**Figure 5:**
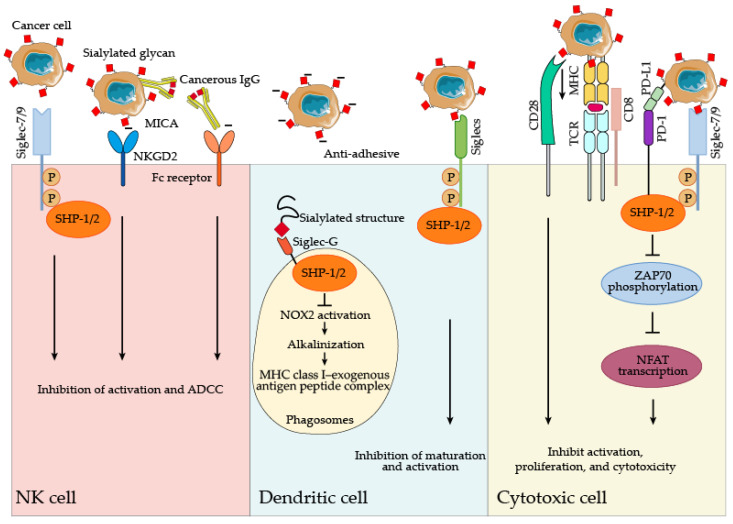
The impact of sialylation on immune cell functions. Siglec: sialic acid-binding immunoglobulin-like lectin; MICA: MHC class I–related chain A; NKGD2: natural-killer group 2, member D; IgG: immunoglobulin G; ADCC: antibody-dependent cell-mediated cytotoxicity; SHP: Src homology region 2 domain-containing phosphatase; MHC: major histocompatibility complex; TCR: T cell receptor; PD-1: programmed death-1; PD-L1: programmed death-ligand 1; ZAP70: zeta chain of T cell receptor associated protein kinase 70; NFAT: nuclear factor of activated T-cells.

Beyond masking effects, the terminal sialic acid-containing glycans (sialoglycans) attached by sialylation can be recognized by the types of immune cells to modulate immune functions (Figure 5). Sialylation of MHC class I–related chain A (MICA) masks its interaction with the activating NK cell receptor, natural-killer group 2, member D (NKG2D), and induces low intrinsic immunogenicity [116,117]. Cancerous IgG has sialylated N-glycans at both Fc and Fab fragments, whereas the classic IgG only exhibited sialylated N-glycans at Fc fragments [118]. Sialylated cancerous -IgGinhibits the antibody-dependent cell-mediated cytotoxicity (ADCC) of NK cells [119] and the proliferation of effector T cells [118]. Moreover, sialylated tumor-associated carbohydrate antigens such as sT and sTn antigens impair DC maturation and limit its capacity to trigger anti-tumor T-cell responses [116,120]. The decrease in tumor-specific T-cell responses is also induced by the internalization of sialylated antigens and the reduction of DC co-stimulatory molecule expression and cytokine secretion caused by sialylated gangliosides [121,122]. Thus, abnormal sialylation confers the immunosuppressive status to cancers. Additionally, interactions of sialoglycans and sialic acid-binding immunoglobulin-like lectins (Siglecs) are considered glyco-immune checkpoints for cancer [123,124,125,126]. α2,3-Sialylated glycans on tumor cells dictate the differentiation of monocytes into immunosuppressive macrophages through Siglec-7 and Siglec-9 signalings [127,128]. Tumor cells’ sialylated glycans also shield them from NK-mediated killing by recruiting Siglec-7 on NK cells [129,130,131]. Moreover, these tumor-expressed sialoglycan ligands interact with Siglec-5, Siglec-9, and Siglec-15 on T cells, suppressing TCR-mediated signaling pathways and corresponding effector functions [132,133,134,135,136]. The binding of α2,3-linked sialic acids to Siglecs modulates the immunosuppressive phenotype of lipopolysaccharide-matured DC by decreasing the phosphorylation of molecules in the JAK-STAT pathways [137].

Additionally, tumor-secreted sialylated gangliosides inhibit DC co-stimulatory molecule expression, cytokine secretion, and T cell proliferation [122]. Sialylated antigens internalized by DCs can interact with Siglec-G on DC phagosomes, hindering the formation of the MHC class I–peptide complex, thus inhibiting DC cross-presentation [138]. As a result, blocking the sialic acid-Siglec axis could be an effective immunotherapeutic strategy against cancer.

## 6. Sialylation and Anti-Cancer Therapy

According to the foregoing research, sialylation is essential for tumor metastasis and anti-cancer immunity. Targeting aberrant sialylation and its ligand-receptor interaction are promising therapeutic strategies for cancer. Previous studies have demonstrated that removing sialic acids on tumor cell surfaces using neuraminidase improves tumor immunogenicity [107,108,139,140,141,142,143]. Since then, efforts have been made to develop treatments for cancer that focus on disrupting sialic acid metabolism and related signaling. Inhibitors of STs and selectins, antibodies targeting selectins and Siglecs, antibody-sialidase conjugates, and glycan vaccines are examples of existing anti-cancer strategies [4].

### 6.1. Blocking Sialic Acid Metabolism

Sialic acids decorate glycoproteins and glycolipids on the surfaces of tumor cells and are engaged in a variety of tumor-related biological functions. Anti-cancer strategies that interfere with the formation of sialoglycans are possibly applicable. STs are key enzymes in sialoglycan biosynthesis. Most ST inhibitors are analogs of sialic acids and CMP-sialic acids [144,145]. 3Fax-Peracetyl Neu5Ac (P-3Fax-Neu5Ac) is a mimetic of sialic acid serving as a global sialyltransferase inhibitor. Adjusting the modifications at C-5 enhances the inhibitor’s potency [146]. The treatment of cancer cells with P-3Fax-Neu5Ac inhibits their capacity for migration and proliferation [11,68]. According to in vivo investigations, this ST inhibitor can enhance cytotoxic CD8+ T cell-mediated anti-tumor response and limit tumor development in a variety of tumor types [147,148,149].

Other ST inhibitors have also been investigated. Soyasaponin I is a potent ST inhibitor that inhibits the cellular ST3Gal activity preferentially, resulting in decreased tumor cell invasiveness [150]. 8-Keto-sialic acid terminates the elongation of α2,8-linked sialic acid chains (oligo- and polysialic acid) [151]. Furthermore, endogenous 5-methyl CMP impairs CST transport and disrupts sialic acid metabolism [152]. Since sialic acid metabolism is a critical biological process, the consequent adverse effects of inhibiting this process deserve consideration.

### 6.2. Interfering with Ligand-Receptor Interaction

Sialic acids’ ligand-receptor interactions with selectins and Siglecs are crucial to the biological behavior of cancer. Blocking E-selectin with Uproleselan significantly affects the extravasation and adhesion of tumor cells, limiting tumor metastasis [153]. Chemotherapy for docetaxel-resistant prostate cancer benefits from P-selectin targeting with Fucoidan [154]. A glycopolymer with a strong affinity for P-selectin is intended to prevent melanoma spread in a mouse model [155]. These inhibitors also inhibit the E-/P-selectin-mediated signaling of tumor cells, leading to additional anti-cancer effects.

The interactions between Sialic acid and Siglecs are considered glyco-immune checkpoints for cancers [123,124,125,126]. Antibodies against Siglec-7 and Siglec-9 augment anti-cancer immunity by inhibiting the polarization of macrophages into tumor-associated macrophages (TAMs) and reprogramming the immunosuppressive tumor microenvironment [156]. Antibodies against Siglec-9 suppress the induction of inhibitory intratumoral cytotoxic CD8+ T cells by Siglec-9, hence boosting TCR signaling, cytotoxicity, and cytokine production of CD8+ T cells [132,133]. Targeting Siglec-15 with antibodies decreases Siglec-15-mediated T cell suppression, thereby enhancing anti-tumor immunity and limiting tumor development [135,157]. The blocking antibodies for Siglec-7, Siglec-9, and Siglec-15 exhibit the ability to restore anti-tumor immunity and reduce tumor burden in vivo, making them potential alternative treatments for cancer patients resistant to the well-known PD-L1/PD-1 blockades [132,133,135,156,157].

### 6.3. Antibody-Sialidase Conjugates

The glycosylation of PD-L1 reduces the efficacy of blocking antibodies against it. Artificial PD-L1 antibody–sialidase conjugate enhances blocking efficiency and desialylates tumor cell surface, hence augmenting anti-cancer activity via T-cell reactivation [158]. The HER2 antibody–sialidase conjugate also increases antibody affinity and destroys tumor-derived sialoglycans, thus boosting anti-tumor immune response and extending the survival of tumor-bearing animals [105]. E-602 is a bi-sialidase fusion protein that targets immunosuppressive sialoglycans. GLIMMER-01 (glycan-mediated immune regulation, NCT05259696) study will investigate its safety, pharmacodynamic effects, and antitumor activity in patients with advanced cancer. This clinical trial represents a significant milestone in the release of immune suppression by modifying cancer cell surface glycans and in the battle to overcome immune resistance in cancer [159].

Intriguingly, the ligand effects of sialic acids can be utilized in the development of drug delivery systems. The sialic acid-cholesterol conjugate liposomal vehicle has been reported to be an effective targeting drug delivery strategy [160,161]. Chemoimmunotherapy is enhanced by the administration of doxorubicin, metformin, and anti-PD-1 monoclonal antibody using this sialic acid-based liposomal platform [160]. This drug delivery system also increases the uptake of epirubicin by TAMs, resulting in the depletion of TAMs and the suppression of tumor development [161]. Generally, utilizing sialic acid-Siglec interactions, strategies that interrupt sialic acid/sialoglycan biosynthesis, block Siglecs, and selectively deliver anti-cancer medicines to Siglec-positive cells are being developed. For the individual patient, it is essential to select the most appropriate strategies from among these numerous options.

### 6.4. Vaccines Development

Altered glycosylation is a common feature of tumor cells and leads to the formation of tumor-associated carbohydrate antigens [162]. Aberrant expression of glycan structures, as well as novel structures of glycan such as sialoglycans, are often accompanied by the expression of tumor antigens [163,164]. Accordingly, researchers have developed carbohydrate-based cancer vaccines (including sialylated epitopes) that stimulate IgG and/or IgM responses in ovarian cancer patients [165,166]. As a tumor-expressed sialylated ganglioside, GM3 is an attractive target for developing therapeutic cancer vaccines despite its low immunogenicity. The barrier is overcome by modifying the sialic acid residue, and the resultant GM3 derivatives are regarded as promising vaccine candidates [167]. Similarly, the antigenicity of sTn is improved by the modification of the sialic acid residue. And these sTn-based glycoconjugates show great promise as vaccination strategies due to their ability to elicit high titers of antigen-specific IgG antibodies [168]. A synthetic STn–keyhole limpet hemocyanin (KLH) vaccine (Theratope^®^) is shown to improve the triple survival rates of advanced breast cancer in phase II clinical trials [169]. Moreover, relative carbohydrate vaccines (a pentavalent vaccine, Globo-H–GM2–sTn–TF–Tn, and a heptavalent vaccine, GM2–Globo-H–Lewis Y–Tn–sTn–TF–Tn-MUC1) are reported to elicit IgG and/or IgM responses in ovarian cancer patients [165,166]. However, cytotoxic T lymphocytes may develop different recognition epitopes for glycosylated and nonglycosylated peptides. A nonglycosylated vaccine for MUC1 can only recognize the nonglycosylated peptide but not tumor-associated MUC1 that is aberrantly glycosylated. Finally, immunological recognition of tumor-associated MUC1 is achieved using a completely manufactured aberrantly glycosylated MUC1 tripartite vaccine [114]. Interestingly, in our earlier investigation, desialylated whole-cell tumor vaccines were able to induce T-cell clones to recognize and kill parental tumor cells in a DC-dependent manner [170]. Thus, the vaccine strategies based on the protein backbone might limit to elicit effective anti-tumor immune responses. Potential solutions may lie in the modification of intricate glycan structures, especially sialoglycans.

## 7. Conclusions

Our findings indicate that current cancer sialylation research focuses on metastasis and immunotherapy. The aberrant sialylation of integrins is a primary mechanism by which tumor cells activate endothelial cells and disseminate to distant organs. Immunologically speaking, cancer sialylation conceals tumor antigen epitopes and develops an immunosuppressive milieu, allowing cancer cells to evade immune surveillance. Targeting tumor-derived sialoglycans is therefore a prospective cancer therapy strategy for preventing the spread of tumor cells, exposing immunogenic tumor antigens, and enhancing anti-cancer immunity. The identification of exact tumor sialoglycans, glycoproteins, or glycolipids could drive the discovery of prospective glycan targets and pave the way for patient-tailored cancer treatments.

## Figures and Tables

**Table 1:** Top 10 articles with the highest citations of cancer sialylation articles.

Rank	Title	First Author (Year)	Source	Citations *
1	ST6Gal-I protein expression is upregulated in human epithelial tumors and correlates with stem cell markers in normal tissues and colon cancer cell lines [10]	Swindall AF et al. (2013)	Cancer Research	52
2	Sialylation of epidermal growth factor receptor regulates receptor activity and chemosensitivity to gefitinib in colon cancer cells [19]	Park JJ et al. (2012)	Biochemical Pharmacology	49
3	Phenylboronic acid-installed polymeric micelles for targeting sialylated epitopes in solid tumors [21]	Deshayes S et al. (2013)	Journal of the American Chemical Society	47
4	Interactions between Siglec-7/9 receptors and ligands influence NK cell-dependent tumor immunosurveillance [15]	Jandus C et al. (2014)	The Journal of Clinical Investigation	39
5	High-throughput profiling of protein N-glycosylation by MALDI-TOF-MS employing linkage-specific sialic acid esterification [17]	Reiding KR et al. (2014)	Analytical Chemistry	37
6	Targeting aberrant sialylation in cancer cells using a fluorinated sialic acid analog impairs adhesion, migration, and in vivo tumor growth [11]	Bull C et al. (2013)	Molecular Cancer Therapeutics	34
7	ST6Gal-I sialyltransferase confers cisplatin resistance in ovarian tumor cells [20]	Schultz MJ et al. (2013)	Journal of Ovarian Research	32
8	Acute myeloid leukemia stem cells and CD33-targeted immunotherapy [16]	Walter RB et al. (2012)	Blood	31
9	Anti-CD22-chimeric antigen receptors targeting B-cell precursor acute lymphoblastic leukemia [22]	Haso W et al. (2013)	Blood	30
10	N-linked glycan structures and their expressions change in the blood sera of ovarian cancer patients [18]	Alley WR et al. (2012)	Journal of Proteome Research	29

* This table was generated automatically by analyzing the articles retrieved on sialylation in cancers using the bibliometrix package in R version 4.2.0. The articles were retrieved from the Web of Science (WoS) Core Collection on 12 September 2022, using the following search strategy: (TS=(sialic acid OR sialylation OR sialoglycan)) AND (TS=(cancer OR tumor OR malignancy OR carcinoma OR neoplasm)). The article-type publications between 2012 and 2021 retrieved were filtered for the final analysis. The top 10 articles listed in Table 1 were the “Most_Local_Cited_Documents. Citation shows the frequency cited by these retrieved ariticles.
